# Introduction to the *JCTS* special issue on advancing understanding and use of impact measures in implementation science

**DOI:** 10.1017/cts.2025.10227

**Published:** 2026-01-07

**Authors:** Kathleen R. Stevens, Julio C. Facelli

**Affiliations:** 1 School of Nursing, Institute for Integration of Medicine & Science, University of Texas Health San Antonio, San Antonio, TX, USA; 2 Department of Biomedical Informatics, School of Medicine and Utah Clinical and Translational Sceince Institute, The University of Utah, Salt Lake City, UT, USA

## Beyond metrics: defining impact in implementation science

Implementation science stands at a pivotal crossroads—and the decisions we make now will shape the future of our field. As our discipline matures, the imperative is no longer simply to move evidence into practice, but to measure and demonstrate real-world impact with clarity and rigor. We believe that the next stage of implementation science demands urgent action: We must capture and communicate meaningful impact across health systems, communities, and policy, or risk falling behind in a rapidly evolving research landscape.

## The urgency is real

Stakeholders (including NIH study sections, federal agencies, private foundations and community partners) are already demanding clear evidence of real-world impact from implementation research. The use of validated impact measures such as those included in the Translational Science Benefits Model (TSBM) is no longer optional; it is a requirement for demonstrating accountability, justifying public investment, and sustaining research funding. In today’s competitive funding climate, researchers must show how their work translates into tangible benefits for patients, communities, and health systems. If we do not act now to adopt robust impact measures, our field faces diminished credibility, reduced funding opportunities, and weakened partnerships with those we aim to serve.

Measuring impact is not just an academic exercise, it is the foundation for accountability, learning, and progress. This themed issue of the *Journal of Clinical and Translational Science* marks a critical inflection point. Building on the foundation laid in the 2020 special issue on dissemination and implementation science, our editorial team advances the conversation by focusing on quantitative impact measures, especially the TSBM. The TSBM was developed to assess and showcase the broader benefits of translational research, moving beyond traditional metrics like publications, number of trainees and grants to include clinical, community, economic, and policy domains. Its evolution and adoption across CTSA hubs and research frameworks reflect the growing recognition that our field must demonstrate tangible and measurable value to stakeholders and society—now more than ever.

Yet, the TSBM’s journey is more than a technical advance; it is a mirror for the field itself. Its expansion, adaptation, and integration into diverse settings reveal a discipline striving to align research with real-world needs, equity, and societal benefit. Implementation science is uniquely positioned to bridge the gap between research and practice. However, to fulfill this promise, our field must move beyond traditional process metrics and urgently embrace impact measures that measure meaningful change. The TSBM and similar frameworks provide tools to assess not only whether interventions are implemented, but also whether they produce lasting improvements that impact on all aspects of human health (clinical, community, prevention, economic, and policy).

As highlighted by Emmons et al., established measures of implementation outcomes can be reframed as precursors to broader impact, making it essential for implementation researchers to integrate these measures into their work. If we fail to do so, we risk losing momentum and relevance in an environment where demonstrating value is paramount.

## Synthesis of themes and article highlights

The articles in this special issue collectively advance our understanding of impact measurement in implementation science. They span key themes: Equity as a central imperative, deep community engagement, pragmatic real-world implementation strategies, capacity building and workforce development, and the expansion of measurement domains to include behavioral economics and organizational readiness. Together, these contributions illustrate the flexibility of the TSBM and related models, showing how they can be adapted to diverse contexts and needs and reinforcing the importance of aligning implementation efforts with measurable, meaningful outcomes reflecting the value proposition of translational science.

## Looking forward: a call to action

The future of implementation science depends on our ability to rigorously measure and communicate impact. There is an urgency for us to advance frameworks like the TSBM to reflect the complexities of implementation research, prioritize equity in every evaluation, and invest in infrastructure that supports ongoing capacity building. By doing so, we will not only strengthen the field of implementation science credibility but also enhance our ability to partner with communities, secure funding, and drive meaningful changes in health systems toward the goal of effective, efficient care for everyone everywhere.

The goals for collective action include the following:

- **Refine and validate impact frameworks**. For example, the adaptation of the TSBM in the Centers for Diabetes Translation Research (CDTR) Impact Framework demonstrates how models can be tailored to capture nuanced outcomes in diabetes care and prevention. We encourage teams to pilot, test, and refine frameworks in diverse settings (e.g., cancer control, hypertension, and primary care) ensuring they resonate with stakeholders and reflect true societal benefit.

- **Center equity in every measure**. The work by Huebschmann and colleagues shows how empirical evidence and logic model refinements can make equity a central criterion. We call on researchers to adapt their impact models to confront disparities in access, outcomes, and community resources, such as integrating Indigenous knowledge and prioritizing underserved populations.
- **Deepen community engagement**. The case example of community partner co-authorship and the use of Community Advisory Boards (CABs) in primary care settings illustrate the power of genuine partnership. We urge institutions to co-create implementation strategies with stakeholders and community members, ensuring that interventions reflect lived experience and local complexity.
- **Invest in infrastructure and workforce development**. The integration of TSBM into mentored training programs and the emphasis on capacity-building in cancer centers highlight the importance of robust institutional support. We advocate for sustained investment in training, mentorship, and infrastructure to build readiness for evidence-based practice at every level.
- **Expand measurement domains and time horizons**. Articles in this issue broaden the scope of impact measurement to include behavioral economics, organizational readiness, and prevention science. For instance, the use of rapid cycle approaches and cost analyses in pragmatic implementation studies enrich our understanding of what matters in real-world settings. We encourage researchers to explore new domains and consider the long-term, temporal dimensions of impact.

## Our charge

We call on researchers, funders, institutions, and community partners to join us in advancing this agenda. Let us continue to test and refine existing frameworks and pilot new frameworks in diverse health conditions, adapt models to prioritize equity, co-create strategies with communities, invest in workforce development, and broaden our measurement horizons to the business model of translational science. Together, we can ensure that implementation science not only drives measurable change but also transforms health systems, communities, and lives.

Our collective commitment will define the next era—one where impact is not just measured but truly realized.

Let us move beyond metrics. Let us demonstrate impact.

